# Study on the rooting promotion of chrysanthemum cuttings by *Massilia consociata* KC 009

**DOI:** 10.3389/fpls.2025.1685038

**Published:** 2025-10-08

**Authors:** Chunmei Lu, Haotian Dong, Yu Jiao, Murad Muhammad, Yuanchun Yang, Jing Zhang, Rui Liu, Yanru Cao

**Affiliations:** ^1^ College of Agriculture and Life Sciences, Kunming University, Kunming, China; ^2^ Institute of Agricultural Sciences, Tacheng Prefecture, Ili Kazakh Autonomous Prefecture, Xinjiang Uyghur Autonomous Region, China; ^3^ State Key Laboratory of Desert and Oasis Ecology, Key Laboratory of Ecological Safety and Sustainable Development in Arid Lands, Xinjiang Institute of Ecology and Geography, Chinese Academy of Sciences, Urumqi, China; ^4^ University of Chinese Academy of Sciences, Beijing, China

**Keywords:** *Massilia consociata*, chrysanthemum cuttings rooting, soluble protein, endophytic bacteria, functional prediction

## Abstract

Continuous cropping reduces the rooting rate and quality of chrysanthemum cuttings, negatively impacting the development of the chrysanthemum industry. This study provides the first evidence that *Massilia consociata* enhances root formation in chrysanthemum cuttings. Using pot experiments, combined with physiological indicator measurements and analysis of the endophytic microbial composition of the chrysanthemum cuttings, the mechanism promoting the rooting effect was investigated. After 10^6^ CFU/mL of KC 009 fermentation was applied to chrysanthemum cuttings for 21 days, the rooting rate, root number, root length and root dry weight significantly increased by 28.97%-50% (*p* < 0.01). Some physiological indicators such as soluble protein, soluble sugar, chlorophyll and indole-3-acetic acid (IAA) were significantly enhanced. Correlation analysis between rooting and physiological indicators revealed that soluble protein was the most critical physiological factor contributing to root formation. The results of high-throughput sequencing of rhizosphere and endophytic microorganisms in chrysanthemum cuttings showed that KC 009 significantly reduced the richness and diversity of endophytic microorganisms. The dominant endophytic bacteria changed from *Ochrobactrum* to *Chryseobacterium* and *Alcaligenes*, which could produce IAA and enhance plant stress resistance. Spearman correlation analysis showed that *Chryseobacterium* was significantly positively correlated with soluble protein, starch, and chlorophyll, and *Alcaligenes* was positively correlated with PPO, POD, and soluble sugar. The abundance of *Cladosporium*, a potential pathogen in endophytic fungi, decreased by 16.70% (*p* < 0.05). Mantel test analysis indicated that soluble protein and starch were most closely related to the endophytic bacterial and fungal communities of chrysanthemum, respectively. Functional prediction of endophytic bacteria revealed that the abundance of 14 metabolic pathways related to plant growth was significantly increased. This study provides theoretical and practical references for promoting the rooting of chrysanthemum cuttings, holding significant importance for the development of the chrysanthemum industry.

## 1 Introduction

Chrysanthemum is one of the world’s four cut flowers along with rose, carnation, and gladiolus (Liu et al., 2023). Beyond its ornamental value, the chrysanthemum is rich in cultural connotation. It is honored as the “gentleman of flowers” in China. It is commonly used in celebrations and ceremonies, representing good fortune and longevity in many countries (Islam et al., 2021). Furthermore, chrysanthemum contains various bioactive compounds, including flavonoids, terpenoids, and polysaccharides, which contribute to its medicinal properties, such as an antihypertensive effect (Zhou et al., 2024), lipid-lowering, anti-inflammatory (Liang et al., 2017), and antibacterial activity (Wang et al., 2022). The wide range of application value makes the market demand for chrysanthemum continues to grow.

Chrysanthemum has diverse methods of propagation, including seed propagation, division, tissue culture, and cutting propagation. Cutting propagation is the most commonly used method for chrysanthemum cultivation due to its advantages, including the preservation of the genetic traits of the mother plant, low cost, simple operation, and a high propagation coefficient (Gao et al., 2023). However, with the increase in cultivation years, autotoxic substances such as coumaric acid gradually accumulate, and the microbial community also changes, which significantly inhibits the survival rate, rooting ability, and growth of chrysanthemum cuttings (Liao et al., 2024), greatly limiting the market supply of chrysanthemums. Currently, the improvement of rooting efficiency in chrysanthemum cuttings is primarily achieved through the application of chemicals, including growth regulators (Kaur and Jhanji, 2023), nutrient solutions (Quevedo et al., 2022), and phytohormones (Joel et al., 2023). However, long-term use and residual accumulation of these chemical substances may disrupt the balance and function of soil micro-ecosystems (Gupta et al., 2017). Therefore, it is necessary to explore green and low-risk alternative strategies to reduce dependence on chemical agents. Soil microorganisms are core members of the soil ecosystem and are closely associated with the healthy growth of plants. Plant Growth-Promoting Rhizobacteria (PGPR) can directly promote plant growth by producing growth hormones and activating other nutrients (Mohanty et al., 2021). They can also indirectly promote plant growth by suppressing plant pathogens, degrading autotoxic compounds from continuous cropping, and improving the rhizosphere microecological environment (Oleńska et al., 2020). These functions make PGPR a significant breakthrough in promoting the rooting and growth of plants. At present, the growth-promoting and disease-resistance effects of many PGPR have been widely verified (Shilpa et al., 2024).

The genus *Massilia* was first reported in 2000 (Kämpfer et al., 2011). Subsequent studies have shown that *Massilia* is widely distributed in the rhizosphere of various plants and exhibits plant growth-promoting traits such as nitrogen fixation, phosphate solubilization, production of plant hormones (Holochová et al., 2020), siderophore production (Diettrich et al., 2019), and ACC deaminase activity (Behrangi et al., 2024). *Massilia niastensisi*, which was isolated from potato roots, can generate indole-3-acetic acid (IAA) and degrade cellulose. The strain can significantly boost the tuber growth of *Solanum tuberosum* (Turnbull et al., 2012). *Massilia* sp. Sco-D23, isolated from the rhizosphere of *Glycine max*, was found to activate the glycolytic pathway in *G. max*, thereby promoting the accumulation of oil in soybean seeds (Han et al., 2024). *Massilia* can not only encourage the growth of plants alone but also synergistically enhance growth when combined with other microorganisms. Krishnamoorthy et al. (2016) inoculated Massilia aerilata together with Rhizophagus intraradices in maize, which enhanced maize growth under salt stress and improved the colonization and sporulation ability of arbuscular mycorrhizal fungi (AMF) at the roots of the maize plant. However, it remains unknown whether *Massilia* possesses root-promoting capabilities.

In our preliminary experiments, *M. consociata* KC 009 was isolated from Jiaozi Mountain in Kunming and identified as being able to promote the rooting of chrysanthemum cuttings. Although studies have reported mechanisms that promote rooting in chrysanthemum cuttings, the role of microorganisms remains unclear, particularly in terms of their effects on physiological indicators of cuttings and the functional dynamics of endophytic and rhizospheric microbial communities. This study employed pot experiments to evaluate the impact of KC 009 on chrysanthemum cutting rooting, aiming to elucidate the mechanism by which *M. consociata* promotes rooting and provide theoretical and practical insights for addressing low rooting rates and poor root quality in chrysanthemum propagation.

## 2 Materials and methods

### 2.1 Experimental materials

The chrysanthemum cuttings used in this study were from the cut-flower cultivar *Chrysanthemum morifolium* Ramat. (‘Jinba’). The cuttings, approximately 8 cm in length with 7–8 leaves, were taken from the healthy apical parts of mother plants, with the basal cut made at a 40-degree angle. Kunming Hongzhihua Horticulture Co., Ltd, provided all samples.

The experimental strain *M. consociata* KC 009 was isolated from the soil of Jiaozi Mountain in Kunming, Yunnan (24°48′N, 102°49′E). The isolation medium was starch-potassium nitrate agar (Seraman et al., 2010), consisting of the following ingredients: soluble starch, 20 g; KNO_3_, 1 g; K_2_HPO_4_·3H_2_O, 0.5 g; MgSO_4_·7H_2_O, 0.5 g; FeSO_4_·7H_2_O, 0.1 g; pH 7. KC 009 was cultured on ISP2 solid medium at 28 °C for 1 day, then transferred to ISP2 liquid medium (Astudillo et al., 2024) and incubated at 28 °C with shaking at 180 rpm for 5 days to obtain the fermentation broth. Some fermentation broth was used for determining the IAA content, while the rest was diluted 1,000-fold (10^6^ CFU/mL) and used for irrigating the chrysanthemum cuttings.

The potting substrate was red soil collected from outside the greenhouse at Kunming University, with a pH of 7.04. The experiment was conducted inside the greenhouse at Kunming University (24°33′N, 102°27′E), Yunnan Province. The average annual temperature is about 15-25°C (Li et al., 2022), and the average sunshine duration is about 6 hours.

### 2.2 Determination of IAA content of KC 009

The fermentation broth of KC 009 was centrifuged at 3000 rpm for 10 min, and the supernatant was collected. The IAA content was determined using a microbial IAA ELISA kit (purchased from JiningBio) and measured at a wavelength of 450 nm with a microplate reader.

### 2.3 Pot experiment design

Stones and other impurities were removed from the red soil substrate, which was then crushed and filled into seedling trays with cells measuring 2 cm × 2 cm × 2 cm. Water the substrate thoroughly the day before planting. Remove 5–6 leaves from each chrysanthemum cutting and insert them into the seedling tray cells at a depth of 1–2 cm, with one cutting per cell. The cuttings were irrigated every three days with the KC 009 fermentation broth diluted 1000-fold. The cultivation period lasted for 21 days. The experiment was conducted at a temperature of 25-28°C, and the humidity ranged from 50% to 80%. Cuttings were sampled every 3 days, and each treatment had three repetitions. Ten chrysanthemum cuttings of each group were chosen at random and stored at 4°C to detect physiological indicators. The control group was watered with the blank medium dilution.

### 2.4 Measurement of chrysanthemum root phenotypes and plant physiological indicators

On the 15th day of cultivation, the rooting rate of cuttings was recorded. Cutting was considered rooted when the adventitious root reached 1 mm. The number of roots was counted, and root length was measured at 21 days. After thorough washing, the roots were washed and fixed at 105°C for 30 min, then dried at 80°C to a constant weight for dry weight determination.

The chlorophyll content of chrysanthemum cuttings was determined by spectrophotometric (Jabborova et al., 2024). The soluble protein content was determined using the Coomassie Brilliant Blue method (Wu and Shen, 2021). Soluble sugar content was determined using anthone colorimetric assay (Zhang et al., 2024), and soluble starch was determined by anthone method (Nayak et al., 2022); IAA and abscisic acid (ABA) contents were quantified with IAA and ABA ELISA kits purchased from Shanghai Youxuan Biotechnology Co., Ltd. Peroxidase (POD) activity was determined by guaiacol method (Nikerova et al., 2023), and polyphenol oxidase (PPO) was determined by catechol method (Song et al., 2023).

### 2.5 High-throughput sequencing of endophytic and rhizosphere microorganisms in chrysanthemum cuttings

After 21 days of cultivation, 0.5 g of rhizosphere soil and 0.5 mg of chrysanthemum cutting tissue, which had been disinfected using 75% alcohol, were collected and stored at –80°C for microbial diversity analysis. Genomic DNA of soil was extracted using the E.Z.N.A.^®^ soil DNA kit (Omega Bio-tek, Norcross, GA, U.S.), In contrast, DNA of chrysanthemum root tissues was extracted using the CTAB method after grinding in liquid nitrogen (Xiao et al., 2024). Samples with DNA purity A260/A280 > 1.8 were used for amplification. The V3–V4 region of the bacterial 16S rRNA gene was amplified using primers 338F (5′-ACTCCTACGGGAGGCAGCAG-3′) and 806R (5′-GGACTACHVGGGTWTCTAAT-3′) (Onyango et al., 2023). The fungal ITS1 region was amplified using primers ITS1F (5′-CTT GGT CAT TTA GAG GAA GTA A-3′) and ITS2R (5′-GCT GCG TTC TTC ATC GAT GC-3 (Liu et al., 2025). PCR reaction was performed in a 20 μL system containing 10 ng of template DNA, 4 μL 5 × Fast Pfu buffer, 2 μL 2.5 mM dNTPs, 0.8 μL each primer (5 μM), 0.4 μL Fast Pfu polymerase, and sterile water added to a final volume of 20 μL. The PCR amplification conditions were as follows: initial denaturation at 95°C for 3 min, followed by 27 cycles of denaturation at 95°C for 30 s, annealing at 55°C for 30 s, and extension at 72°C for 30 s, followed by a final extension at 72°C for 10 min. Three biological replicates were performed for each sample. After verification of the amplicons by 2% agarose gel electrophoresis, a sequencing library was prepared using the Illumina TruSeq^®^ DNA PCR-Free Library Prep Kit. Paired-end sequencing (2 × 270 bp) was performed on the Illumina NovaSeq 6000 platform (Illumina, USA), generating approximately 51,555 high-quality reads per sample. The raw sequencing data were processed on the Majorbio Cloud Platform using Trimmomatic (v0.39) to remove low-quality sequences (Phred score < 20). Paired-end reads were assembled using FLASH (v1.2.11), and chimeric sequences were removed using UCHIME (v8.1). Species annotation was performed based on the bacterial SILVA database (http://www.arb-silva.de) and the fungal UNITE database (http://unite.ut.ee/) with a confidence threshold of 0.7.

### 2.6 Data processing

T-test and ANOVA were performed on experimental data using SPSS 21.0 to evaluate the statistical significance of differences (version 21.0; SPSS Inc, Chicago, IL, USA) (Zhang et al., 2020b). Root phenotypes, physiological indexes, community richness and diversity indices were visualized using Origin 2021 (Moberly et al., 2018). Correlation analysis among physiological indexes, root indexes, and endophytic community composition was performed using (Dag and Ilk, 2017). The analysis and plotting of the intra-endogenous microbial community at the phylum and genus levels were done using Source Tracker software (Rousseau et al., 2020). A diagram of the collinear network of endophytic microbial correlation was plotted with Network (Zhang et al., 2020a). LEfSe was used to analyze changes in endophytic microbial composition (Jin et al., 2022). PICRUSt2 (http://huttenhower.sph.harvard.edu/galaxy) and FUNGuild (http://www.funguild.org/) were used for predictive analysis and mapping of bacterial and fungal functions, respectively (Martínez et al., 2019; Peng et al., 2025).

## 3 Results

### 3.1 Determination of IAA production by KC 009

The microbial IAA ELISA kit was used to detect the fermentation broth of strain KC 009. The results showed that KC 009 could produce 15.995 nmol/L of IAA in ISP 2 liquid medium, indicating that the strain possesses the metabolic capability to synthesize IAA.

### 3.2 Effects of KC 009 on the root morphology of chrysanthemum cuttings

**Figure 1:**
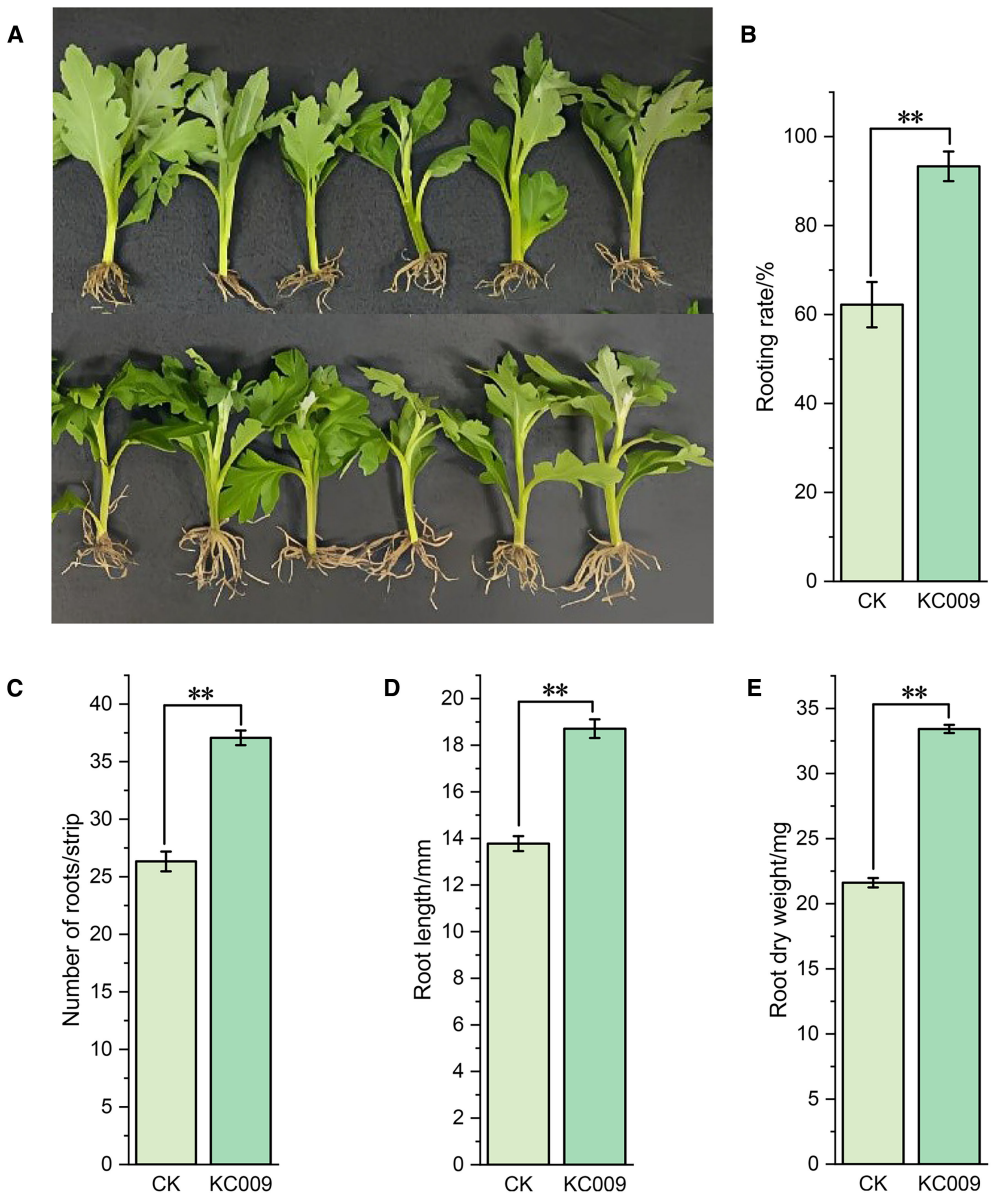
Effect of KC 009 on root growth of chrysanthemum cuttings. **(A)** The root-promoting effect of KC 009 at 21 days. Up, blank treatment; Down, KC 009 treatment. **(B)** Rooting rate at 15 days. **(C)** Number of root at 21 days. **(D)** Root length at 21 days. **(E)** Root dry weight at 21 days. Left, blank treatment; Right, KC 009 treatment. The asterisk represented the significance level. **p < 0.01.

KC 009 application significantly promoted root growth in chrysanthemum cuttings (**Figure 1A**). The statistical analysis on the 15th day of cultivation showed that KC 009 significantly increased the rooting rate of cuttings by 50% (**Figure 1B**). By day 21, the number of roots, root length, and root dry weight of the cuttings increased significantly by 40.78%, 35.77%, and 54.65%, respectively (**Figures 1C-E**). These results indicated that KC 009 had the best effect on improving the rooting rate of chrysanthemum cuttings, followed by enhancements in root length and root dry weight.

### 3.3 Effect of KC 009 on the physiological indexes of cutting chrysanthemums

**Figure 2:**
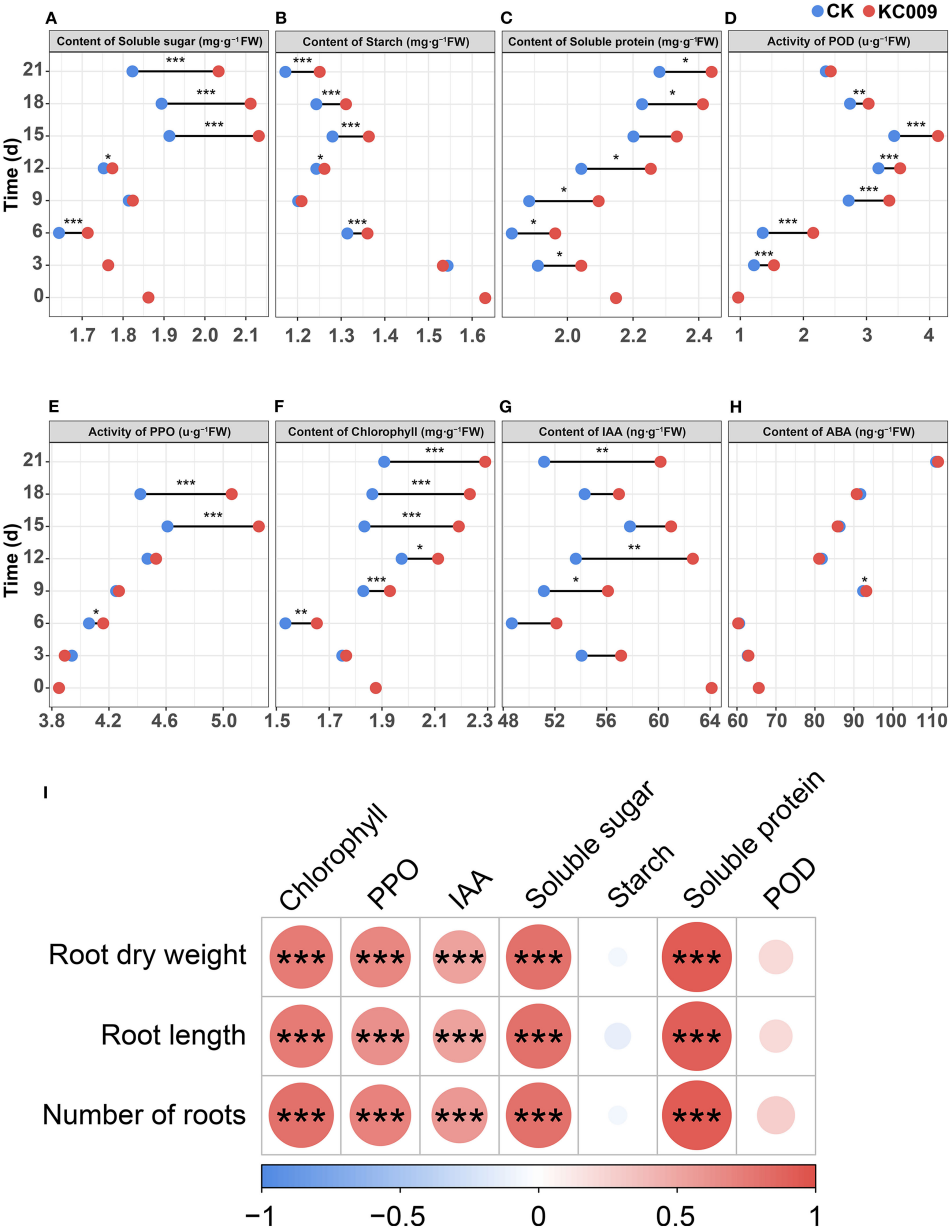
Effect of KC 009 on physiological indexes of chrysanthemum cuttings from 3 to 21 days and theirs correlation with root phenotypic indexes. **(A)** Content of soluble sugar. **(B)** Content of soluble sugar. **(C)** Content of soluble protein. **(D)** Activity of POD. **(E)** Activity of PPO. **(F)** Content of chlorophyll. **(G)** Content of IAA. **(H)** Content of ABA. **(I)** The correlation between physiological indexes and root phenotypic indexes. The asterisks in the graph indicated the significance level. *p < 0.05, **p < 0.01, ***p < 0.001.

After KC 009 treatment, the contents of soluble sugar and starch began to accumulate significantly from day 6, reaching peak increases between days 15 and 21, with significant increases of 11.55% and 6.71%, respectively (**Figures 2A, B**). The content of soluble protein began to rise significantly on day 3 after KC 009 treatment, with a maximum increase of 11.23% (**Figure 2C**). These results indicated that the application of KC 009 promoted the accumulation of carbon and nitrogen substances in chrysanthemum cuttings, and its regulation of different nutrients showed temporal specificity.

The POD activity in chrysanthemum treated with KC 009 was significantly higher than the control group from the third day to the 21st day, with the highest increase occurring on the sixth day, rising by 59.01% (**Figure 2D**). The PPO activity began to increase significantly on the 15th day, with a maximum increase of 14.66% (**Figure 2E**). These results indicated that KC 009 significantly upregulated the POD and PPO activities involved in oxidative damage in chrysanthemum cuttings.

Starting from the 6th day after the application of KC 009, the chlorophyll content in the treatment group began to increase, exhibiting a sharp rise between days 15 and 21, with an increase of 19.44%–19.99% (**Figure 2F**). Regarding IAA, fermentation broth analysis of KC 009 confirmed its ability to produce IAA. From the 3rd day after KC 009 application, the IAA levels in chrysanthemum cuttings significantly increased, showing 16.89% and 17.60% higher levels than the control group on the 12th and 21st days, respectively (**Figure 2G**). This indicated that KC 009 not only synthesizes IAA *in vitro* but also significantly enhances endogenous IAA synthesis in chrysanthemum cuttings. KC 009 treatment had little effect on ABA levels in the cuttings (**Figure 2H**).

Spearman correlation analysis of physiological indexes and root phenotypes revealed (**Figure 2I**) that root dry weight, root length, and root number were significantly positively correlated with soluble protein, soluble sugar, chlorophyll content, and PPO activity, and IAA content, but not significantly correlated with soluble starch and POD activity. These results indicated that the increases of soluble protein, soluble sugar, chlorophyll, and PPO levels were the primary physiological factors contributing to root growth promoted by KC 009, followed by IAA content.

### 3.4 Effects of KC 009 on endophytic microbial diversity in chrysanthemum cuttings

#### 3.4.1 Effects of KC009 on diversity of rhizospheric and endophytic microorganisms in chrysanthemum cuttings

**Figure 3:**
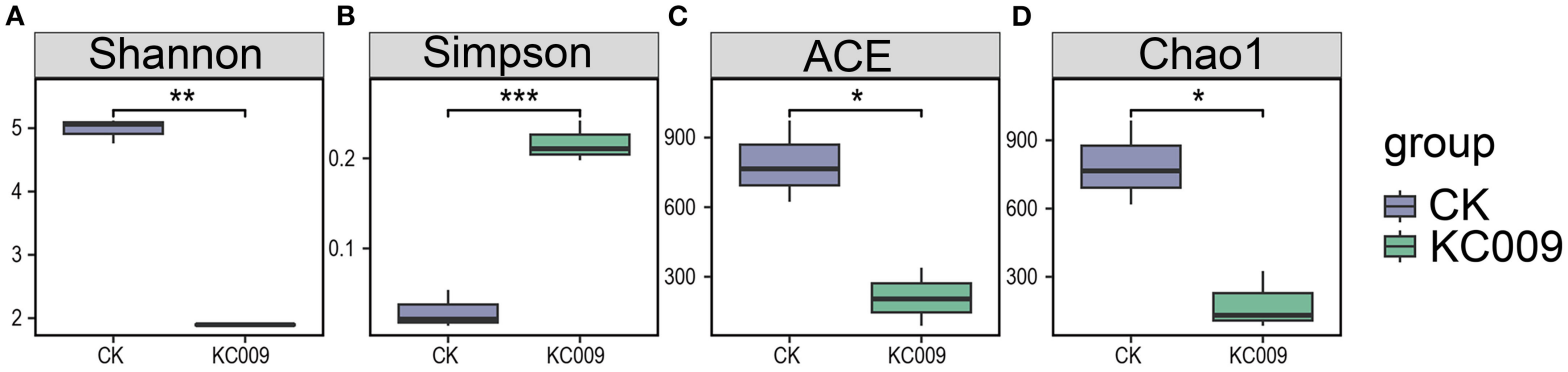
Effect of KC 009 on the diversity of endophytic bacteria in chrysanthemum cuttings. **(A, B)** Shannon and Simpson indices in species diversity. **(C, D)** ACE and Chao1 indices in species richness. The asterisk represented the significance level. *p < 0.05, **p < 0.01, ***p < 0.001.

Alpha diversity analysis of rhizospheric and endophytic microorganisms in chrysanthemum cuttings revealed that KC 009 had no significant impact on the composition or diversity of the rhizospheric microbial community. However, it reduced the richness and diversity of endophytic fungi, while significantly decreasing the richness and diversity of endophytic bacteria (**Figure 3**).

#### 3.4.2 Effect of KC 009 on microbial community composition in chrysanthemum cuttings

**Figure 4:**
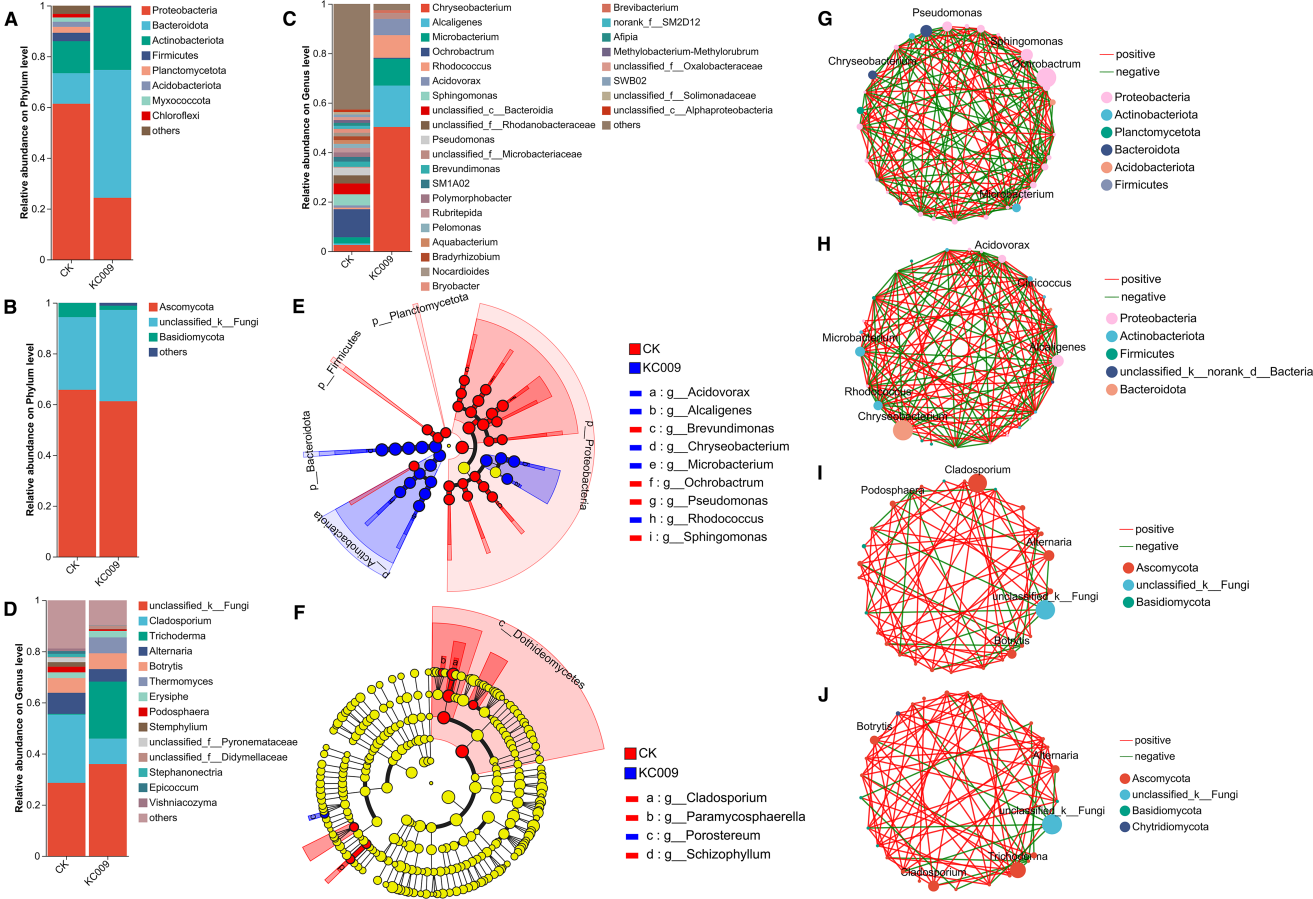
Effect of KC 009 on the endophytic microbial communities. **(A, B)** Community composition at the phylum level. **(C, D)** Community composition at the genus level. **(E, F)** LEfSe analyzed microorganisms with significant differences in abundance. **(G, H)** Co-occurrence network of bacteria. **(I, J)** Co-occurrence network of fungi. **(A, C, E, G, I)**, Bacteria; **(B, D, F, H, J)**, Fungi.

The analysis of endophytic bacterial communities at the phylum level in Chrysanthemum cuttings revealed that KC 009 treatment led to a 37.02% decrease in the relative abundance of Proteobacteria. The abundances of *Bacteroidota* and *Actinobacteriota* increased by 38.33% and 11.94%, respectively. Additionally, the *Planctomycetota*, *Acidobacteriota*, and *Chloroflexi* disappeared (**Figure 4A**). At the genus level, KC 009 significantly increased the relative abundances of *Chryseobacterium*, *Alcaligenes*, *Rhodococcus*, *Microbacterium*, and *Acidovorax* by 45.68%, 17.98%, 9.96%, 6.62%, and 6.01%, respectively (**Figure 4C**). LEfSe analysis (LDA score ≥ 4.0) showed that *Chryseobacterium* had the highest LDA score of 5.35, followed by *Alcaligenes* with a score of 4.90, indicating that these two genera are significant biomarkers distinguishing the KC 009 treatment group from the control group (**Figure 4E**). Co-occurrence network analysis of the top 30 most abundant endophytic bacteria revealed that the network density of bacterial associations increased from 0.35402 to 0.37566 after KC 009 treatment, suggesting that KC 009 enhanced the interactions and stability of the endophytic bacteria in chrysanthemum cuttings. Simultaneously, the significantly enriched *Chryseobacterium* showed positive correlations with beneficial plant growth-promoting genera such as *Sanguibacter*, *Kocuria*, and *Microbacterium* (**Figures 4G, H**).

Regarding endophytic fungi, KC 009 treatment reduced the abundance of *Ascomycota* and *Basidiomycota* at the phylum level by 4.51% and 3.90%, respectively (**Figure 4B**). At the genus level, KC 009 led to a 26.67% increase in *Trichoderma* and a 9.77% increase in *Thermomyces* (**Figure 4D**), while reducing the relative abundances of potential pathogens *Cladosporium* and *Alternaria* by 16.70% and 3.35%, respectively. Furthermore, several fungal genera including *Botrytis*, *Podosphaera*, *Stemphylium*, *Epicoccum*, *Acremonium*, *Meyerozyma*, and *Stephanonectria* were completely eliminated following KC 009 treatment. LEfSe analysis revealed that the most significant biomarker shifted from *Cladosporium* to *Proteosporum* after KC 009 treatment (**Figure 4F**). Co-occurrence network analysis of the top 30 most abundant endophytic fungi revealed that the number of connections between network nodes increased from 152 to 168, and the network density rose from 0.18719 to 0.20689, suggesting that KC 009 enhanced the species richness and stability of the endophytic fungi in chrysanthemum cuttings. Furthermore, *Cladosporium* showed a positive correlation with potential pathogenic fungi such as *Ramularia*, *Erysiphe*, and *Podosphaera* in the control group (**Figure 4I**). In contrast, the abundance of *Trichoderma* increased and showed a positive correlation with the probiotic *Acremonium* in the KC 009 treatment group (**Figure 4J**).

In summary, KC 009 enhanced the interactions and stability of the endophytes in chrysanthemum cuttings. As the bacterial and fungal biomarkers, *Chryseobacterium* and *Proteosporum* contributed the most to the inter-group differences in the endophytic microbial community after KC 009 treatment, and both have the potential to promote plant growth.

#### 3.4.3 Correlation analysis between endophytic microbial community and the physiological and root indexes of chrysanthemum cuttings

**Figure 5:**
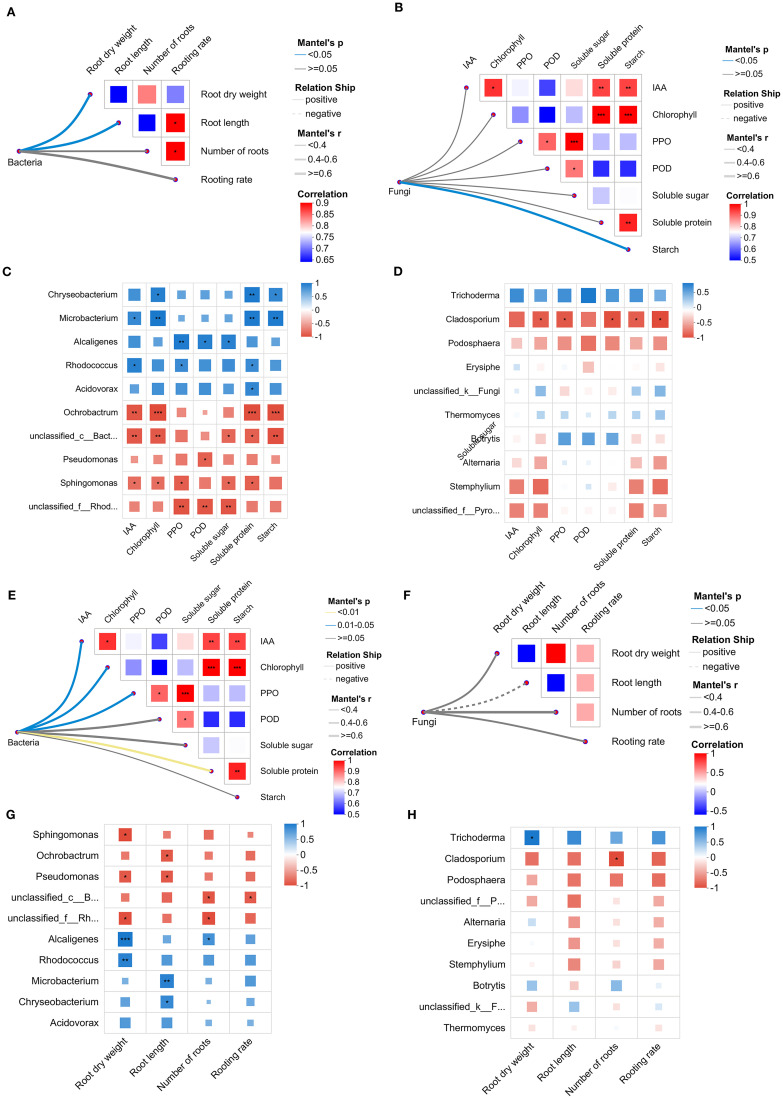
Spearman correlation analysis of endophytic microorganisms with physiological indicators **(A-D)** and root phenotype of chrysanthemums **(E-H)**. **(A, B)** Endophytic microbial community and physiological indicators. **(C, D)** Dominant endophytic genera and physiological indicators. **(E, F)** Endophytic microbial community and root phenotype. **(G, H)** Dominant endophytic genus and root phenotype. Left, bacteria; Right, fungi. The asterisk represented the level of significance. *p < 0.05, **p < 0.01, ***p < 0.001.

Mantel test analysis between endophyte composition and physiological indexes of chrysanthemum cuttings showed that the endophytic bacterial community was extremely significantly positively correlated with soluble protein, and significantly correlated with IAA, chlorophyll, and PPO (**Figure 5A**). In addition, the endophytic bacterial showed a significant positive correlation with root dry weight and root length (**Figure 5E**). Spearman analysis between dominant microbial taxa and physiological indexes revealed that *Chryseobacterium*, whose abundance increased significantly, was positively correlated with soluble protein, and correlated significantly with chlorophyll and starch. The enriched *Microbacterium* exhibited extremely significant positive correlations with chlorophyll, soluble protein, and starch, as well as a significant positive correlation with IAA (**Figure 5C**). Meantime, both *Microbacterium* and *Chryseobacterium* were significantly positively correlated with root length of chrysanthemum cuttings (**Figure 5G**). *Alcaligenes* with significantly increased abundance were positively correlated with physiological indicators, including PPO, POD, and soluble sugars. They were also positively correlated with root dry weight and the number of root.

In contrast, *Ochrobactrum*, whose abundance was extremely significantly reduced, showed highly significant negative correlations with IAA, chlorophyll, soluble protein, and starch. The reduced abundance of *Sphingomonas* showed a significant negative correlation with soluble sugar, soluble protein, IAA, chlorophyll, and PPO (**Figure 5C**). Both *Ochrobactrum* and *Sphingomonas* showed significant negative correlations with root dry weight and root length (**Figure 5G**). The decreased abundance of *Pseudomonas* showed a negative correlation with POD, root dry weight, and root length (**Figures 5C**, **G**).

For endophytic fungi, the whole community was significantly positively correlated with starch (**Figure 5B**). Among them, *Cladosporium*, which exhibited a markedly reduced abundance following KC 009 treatment, showed significant negative correlations with key physiological parameters, including chlorophyll content, PPO activity, soluble sugars, soluble protein, starch (**Figure 5D**), as well as root number. The increased abundance of *Trichoderma* was significantly positively correlated with root dry weight (**Figure 5H**).

### 3.5 Effects of KC 009 on the function and enzyme abundance of the endophytes in chrysanthemum cuttings

#### 3.5.1 Effects of KC 009 on endophytic bacterial community function and enzyme abundance

**Figure 6:**
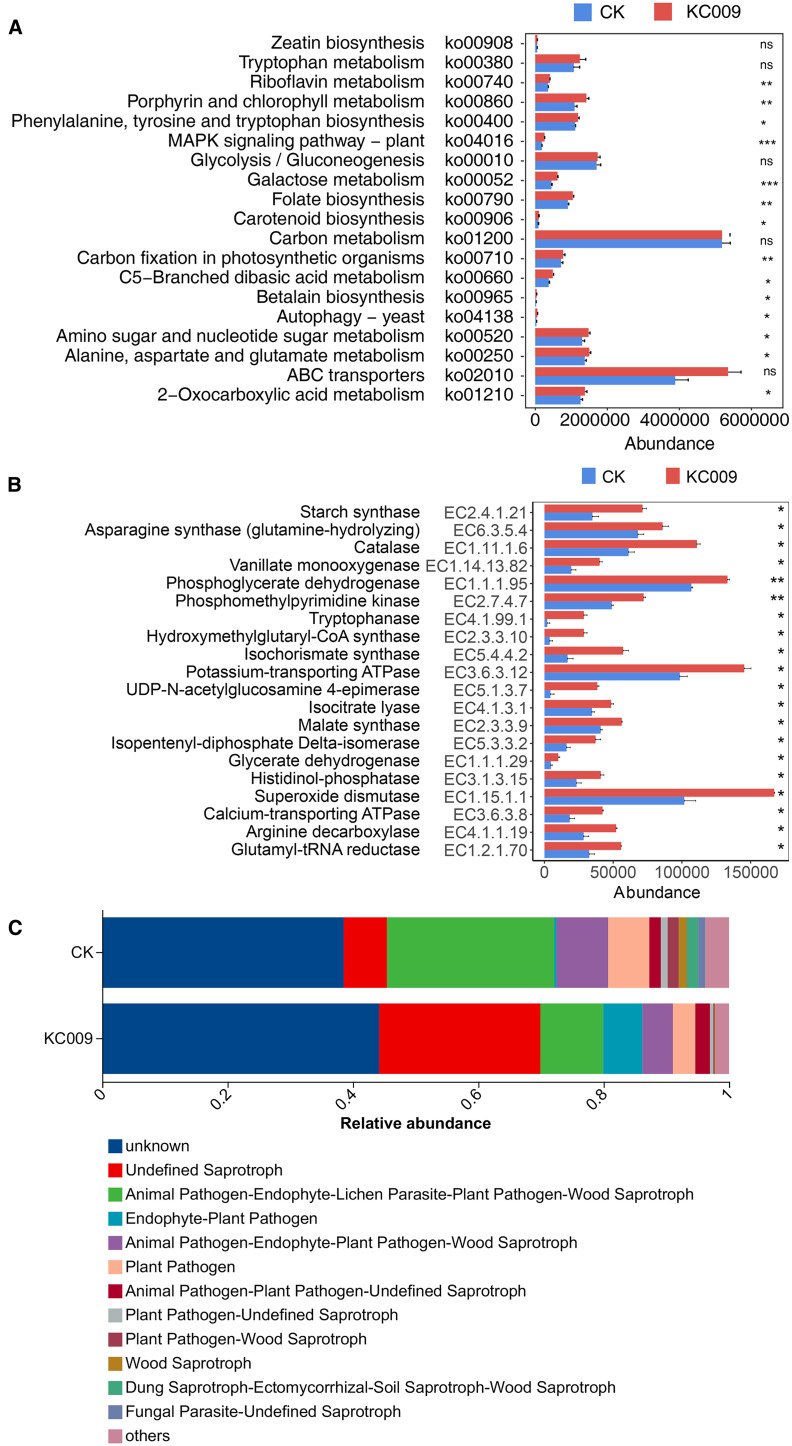
Effect of KC 009 on the function prediction of endophytic bacterial. **(A)** Bacterial function abundance at KEGG Level 3. **(B)** KEGG enzyme abundance of bacteria. **(C)** FUNGuild functional abundance of fungi. The asterisk in the figure represented the significance level, *p < 0.05, **p < 0.01, ***p < 0.001, ns: *p >*0.05.

PICRUSt 2 was used to predict the function of endophytic bacteria in cutting chrysanthemums. The results of KEGG level 2 functional predictive analysis revealed that after treatment with KC 009, the relative abundances of pathways related to amino acid metabolism, carbohydrate metabolism, and energy metabolism significantly increased. This suggested that KC 009 altered the metabolic regulation of amino acids and carbohydrates by endophytic microorganisms in cutting chrysanthemums. KEGG level 3 functional prediction showed that 14 metabolic pathways associated with plant growth were significantly enriched (**Figure 6A**). Among them, the abundance of the folate biosynthesis pathway, MAPK signaling pathway, and galactose metabolism pathway increased significantly by 149.49%, 39.88%, and 38.13%, respectively. The abundance of the C5-Branched dibasic acid metabolism pathway and riboflavin metabolism pathway showed significant increase of 30.44% and 13.56%, respectively.

KEGG Enzyme analysis revealed that 20 enzymes related to plant growth were significantly increased in the KC 009 treatment group (**Figure 6B**). Thereinto, the abundance of Tryptophanase (EC: 4.1.99.1) related to IAA synthesis increased by 129.71%, the abundance of Starch synthase (EC: 2.4.1.21) related to catalyzing starch synthesis significantly increased by 104.44%, and the abundance of Glutamyl-tRNA reductase (EC: 1.2.1.70) involved in plant chlorophyll synthesis increased by 71.34%. The abundance of Catalase (EC: 1.11.1.6), which plays a role in alleviating oxidative stress and enhancing stress resistance, increased significantly by 36.09%. Asparagine is a crucial protein in plants, playing a key role in nitrogen metabolism, energy balance, and stress response (Asif et al., 2022). KC 009 treatment significantly increased the abundance of Asparagine synthase (EC: 6.3.5.4) in endophytic bacteria of chrysanthemum cuttings by 26.37%.

#### 3.5.2 Effects of KC 009 on the ecological function of the endophytic fungal community

Ecological functional analysis of endophytic fungi in cutting chrysanthemums using FUNGuild (**Figure 6C**) revealed that after KC 009 treatment, the abundances of the Animal Pathogen-Endophyte-Lichen Parasite-Plant Pathogen-Wood Saprotroph, Plant Pathogen, Animal Pathogen-Endophyte-Plant Pathogen-Wood Saprotroph were significantly reduced by 62.42%, 45.77% and 40.69%, respectively, and the main fungi of the three functional types were primarily plant pathogens. Additionally, the relative abundance of Undefined Saprotrophs increased by 366.47%, with the main groups being beneficial fungi known to promote plant growth, including *Eurotiales*, *Hypocreales*, *Saccharomycetales*, and *Metarhizium*.

## 4 Discussion

Cutting chrysanthemum is very important in the global floriculture industry, with strong export demand requiring rapid supply and stable quality. The root is a vital organ for nutrient uptake from the soil, and increasing the number and length of roots is essential to enhance nutrient absorption capacity and support the growth and development of the plant. The application of PGPR can not only stimulate root formation and growth in plants, but also alleviate the ecological risk caused by chemical applications. *Massilia*, as a type of PGPR, has not yet been reported to promote rooting in plant cuttings. This study is the first to demonstrate that applying *M. consociata* KC 009 can effectively promote rooting in chrysanthemum cuttings, indicating that *Massilia* can enhance rooting in plant cuttings. This study investigated the effect of *M. consociata* KC 009 on the rooting ability of cut chrysanthemum. It analyzed the potential growth-promoting mechanism of strain KC 009 based on physiological measurements and changes in endophytic and rhizosphere microbial communities. KC 009 promotes the root growth of chrysanthemum cuttings by reshaping the endophyte community and simultaneously enhancing physiological indicators, such as soluble proteins. The correlation analysis between physiological indicators and phenotypic indicators, as well as the correlation analysis between endophyte and phenotypic and physiological indicators of chrysanthemum roots, reveals the physiological indicators and endophytic microbial groups most closely related to chrysanthemum roots. Unlike previous studies that focused solely on a single aspect for promoting root formation, this study elucidates the mechanism by which KC 009 encourages root growth in chrysanthemum cuttings from multiple dimensions.

### 4.1 Significant effects of KC 009 on root development and physiological parameters in chrysanthemum

IAA and ABA play crucial roles in plant growth, development, and environmental adaptation (Feng et al., 2024; Zhang et al., 2019). Song et al. (2025) found that *Bacillus* sp. SYM-4 not only could produce IAA itself but also significantly increased the IAA content of *Zea mays* L. by 77.19% when inoculated. *Massilla*, a representative PGPR, is widely distributed in the rhizosphere soils of various plants. Yao et al. (2024) identified the IAA biosynthesis gene cluster *trpEGDCFB* in *Massilia phyllosphaerae* SCZ-792^T^ isolated from *Pennisetum* sp. They confirmed that the strain can significantly increase primary root length and lateral root number in *Oryza sativa* L. In this study, fermentation of *M. consociata* KC 009 yielded 15.995 nmol/L IAA, and its application to chrysanthemum cuttings significantly increased their endogenous IAA levels by 17.60%. This indicated that KC 009 not only synthesizes IAA *in vitro* but also considerably enhances endogenous IAA synthesis in chrysanthemum cuttings. Furthermore, the rooting rate, root length, number of roots, and root dry weight increased by 50%, 35.77%, 40.78%, and 54.65% respectively.

Soluble sugars, soluble proteins, and soluble starch in plants play essential roles in regulating physiological and metabolic processes, providing nutritional support for growth and development, and enhancing stress resistance (Verma et al., 2024). The application of PGPR could modulate plant physiological activities, thereby promoting root development and growth. Mehrabi et al. (2024) inoculated *Triticum aestivum* with *Bacillus velezensis* UTB96 and found that the starch content of *T. aestivum* increased significantly by 17%-29%. Wyrwicka et al. (2019) inoculated *Massilia niastensis* p87 into *Festuca arundinacea* Schreb. and found that it significantly promoted shoot growth during the later stages of plant development, with a corresponding increase in soluble protein content in the leaves of up to 129%. KC 009 treatment systematically upregulated carbon and nitrogen nutrients, photosynthetic pigment content, and enzyme activity of chrysanthemum cuttings. PPO and POD are important enzymes involved in plant disease resistance defense and stress response. They work together to maintain cellular redox homeostasis and regulate the synthesis of secondary metabolites and strengthen the cell wall, thereby enhancing the plant’s resistance to pathogen invasion and oxidative damage (Zhao et al., 2024). Zhou et al. (2023) found that POD could reduce the adverse effects of oxidative stress on cutting root formation, accelerate wound healing, and promote rooting. In our study, the application of KC 009 significantly increased the POD and PPO content of chrysanthemum cuttings by 59.01% and 14.66%, respectively.

Spearman correlation analysis between root indexes and physiological parameters showed that root growth indexes were significantly positively correlated with increased levels of soluble protein, soluble sugar, chlorophyll, IAA, and PPO activity. These results confirm that the root-promoting effect of KC 009 on cutting chrysanthemum was driven by a coordinated regulatory mechanism involving carbon and nitrogen accumulation, photosynthetic assimilation, hormone signaling activation, and enhancement of related enzyme activities.

### 4.2 Regulatory effects of KC 009 on the rhizosphere and endophytic microbial community of cutting chrysanthemum

The addition of exogenous microorganisms can alter the rhizosphere or endophytic microbial community of plants. Ji et al. (2020) inoculated *Enterobacter cloacae* HG-1 into Wheat cv. Jimai 21, which significantly reduced the diversity and richness of rhizosphere microorganisms, yet significantly increased the root length and plant height. The application of KC 009 had a minimal effect on the rhizosphere microbial composition of chrysanthemum cuttings. Still, it significantly reduced the diversity and richness of the endophytic fungal community, resulting in a reconstructed endophytic bacterial composition. At the phylum level, the relative abundance of *Bacteroidota* and *Actinobacteriota* increased following KC 009 treatment, becoming the dominant bacteria in the endophytic community. Among them, *Bacteroidota* accounted for the most significant proportion in the KC 009 treatment, and its members not only inhibit pathogenic bacteria but also promote the phosphorus absorption and utilization (Lidbury et al., 2021). *Actinobacteriota* are known to facilitate nutrient uptake, thereby promoting plant growth (Oloumi et al., 2023). At the genus level, the abundance of plant growth-promoting bacteria such as *Chryseobacterium*, *Alcaligenes*, and *Microbacterium* significantly increased. *Chryseobacterium*, showing the most significant increase in abundance, was identified as the most important biomarker in the KC 009 treatment. It can secrete IAA, with the potential to stimulate root development. Kumar et al. (2023) found that treating *Arabidopsis thaliana* seeds with *Chryseobacterium cucumeris* PCH239 significantly improved germination rates and promoted primary root and root hair growth. Spearman correlation analysis revealed that *Chryseobacterium* was significantly positively correlated with physiological indicators, such as soluble protein, chlorophyll, and starch, and also positively correlated with beneficial genera, including *Acidovorax* and *Microbacterium*. *Alcaligenes* can promote plant growth by producing ACC deaminase and synthesizing siderophore (Das et al., 2022). Sapre et al. (2021) reported that inoculation with *Alcaligenes faecalis* IG27 significantly increased root length and root dry weight of Pisum sativum by 12.61% and 50%, respectively. *Microbacterium* can promote plant growth by alleviating stress and enhancing the utilization of trace element (Xiao et al., 2025). Zhao et al. (2024) reported that inoculation of *Microbacterium testaceum* M15 into *Oryza sativa* L. significantly increased chlorophyll content by over 30%. In summary, KC 009 promoted root development in chrysanthemum cuttings by enriching beneficial functional microorganisms, optimizing synergistic interactions among microbial communities, and reconstructing a low-diversity yet highly functional microbial community.

For endophytic fungi, *Ascomycota* and *Basidiomycota* were the dominant phyla in both the CK and the KC 009 treatment. Members of *Ascomycota* can promote plant development by regulating carbon and nitrogen cycling (Challacombe et al., 2019). At the genus level, the application of KC 009 significantly suppressed the growth of potential pathogens, such as *Cladosporium* and *Alternaria*, while markedly enhancing the growth-promoting *Trichoderma*. *Trichoderma* can strengthen root development and plant growth by promoting nutrient uptake and secreting growth hormones. *Cladosporium* with significantly reduced abundance often causes leaf spot (Robles et al., 2019) and tomato leaf mold (Yoshida et al., 2020), and is significantly negatively correlated with Chlorophyll, PPO, soluble sugars, soluble protein, and starch. *Alternaria* with reduced abundance not only produces phytotoxins such as Alternariol and AM-toxin to damage plant cell (Andersen et al., 2006), but also causes potato early blight (Patel and Gohel, 2023) and apple black spot (Han et al., 2023). Spearman analysis showed that it was positively correlated with other pathogens, including *Gibberella*, which causes American ginseng root rot disease (Tian et al., 2021), and *Penicillium*, which causes apple rot (Watpade et al., 2025). Co-occurrence network analysis further demonstrated that KC 009 indirectly suppressed the synergistic proliferation of other pathogens by reducing the abundance of *Alternaria*. In conclusion, KC 009 effectively promoted rooting of chrysanthemum through a regulatory effect of “suppressing harm and promoting benefits”. This provides a theoretical basis for developing microbial agents that integrate both plant growth promotion and disease control functions.

### 4.3 Effects of KC 009 on the function and enzyme abundance of endophyte in cutting chrysanthemum

KC 009 significantly enhanced carbon and nitrogen metabolism as well as stress resistance in cutting chrysanthemum by regulating the functional modules of endophytic bacteria. KEGG level 3 analysis showed that KC 009 increased carbohydrate storage in cutting chrysanthemum by upregulating starch synthase (EC: 2.4.1.21), and simultaneously enhancing the abundance of C5-branched dibasic acid metabolism and asparagine synthase (EC: 6.3.5.4) to promote nitrogen accumulation. These findings were consistent with the observed increases of 11.55%, 6.71%, and 11.23% in soluble sugar, starch, and soluble protein content, respectively, in the KC 009 treatment. The application of KC 009 also increased the abundance of glutamyl-tRNA reductase (EC: 1.2.1.70), as well as folate biosynthesis and riboflavin metabolism, thereby synergistically enhancing the synthesis of photosynthetic pigments and contributing to a 19.99% increase in chlorophyll content in chrysanthemum cuttings. The notable increase in tryptophanase (EC: 4.1.99.1) and MAPK signaling pathway-plant abundance was in line with the observed 17.60% rise in IAA content of KC 009 cuttings. A significant increase in catalase (EC: 1.11.1.6) abundance may help eliminate oxidative substances such as H_2_O_2_ during the early stages of callus formation in cutting chrysanthemum, thereby promoting cell division and callus development (Adil et al., 2019).

FUNGuild analysis of endophytic fungi revealed that KC 009 treatment significantly reduced the abundance of Animal Pathogen-Endophyte-Lichen Parasite-Plant Pathogen-Wood Saprotroph, which was predominantly composed of the pathogenic *Cladosporium*. The abundance of the Plant Pathogen also decreased significantly, mainly represented by *Bipolaris* which causing southern leaf blight and brown spot (Bhunjun et al., 2020), and *Cylindrocladiella* associating with root rot, seedling blight, and xylem-related diseases (Li et al., 2021). In addition, the abundance of the Animal Pathogen-Endophyte-Plant Pathogen-Wood Saprotroph, dominated by the pathogen *Alternaria*, was also reduced. These results suggest that KC 009 treatment has the potential to reduce the risk of fungal disease outbreaks in cutting chrysanthemum.

## 5 Conclusions

This study found that *M. consociata* KC 009 not only significantly enhanced physiological indicators, such as POD activity, chlorophyll content, and IAA levels, in cutting chrysanthemum but also reshaped the endophyte community. KC 009 induced a synergistic enhancement of protein and carbohydrate metabolism in endophytes, increased the abundance of starch synthase, chlorophyll synthase, and IAA synthase, and drove the carbon and nitrogen nutrients of chrysanthemum cuttings in the direction of energy storage and auxin synthesis, forming a microecological balance of “inhibiting pathogens and promoting benefit”, thereby significantly increasing rooting rate, root length, and root dry weight by 50%, 35.77%, and 35.33%, respectively. This study revealed the mechanism by which PGPR KC 009 promotes the rooting of chrysanthemum cuttings through a multidimensional synergistic strategy, providing theoretical support and practical reference for the development of microbial agents to regulate the rapid rooting of chrysanthemums. KC 009 can be directly applied to enhance the rooting efficiency of chrysanthemum cuttings, serving as a cost-effective and eco-friendly microbial agent that facilitates rapid rooting in ornamental seedlings during cutting propagation. However, this study is based solely on potted experiments and single chrysanthemum varieties, and it is necessary to supplement this with field verification and applicability tests using more diverse chrysanthemum varieties.

## Data Availability

The original contributions presented in the study are included in the article/supplementary material. Further inquiries can be directed to the corresponding author.
